# Antibiotic Use in Suspected and Confirmed COVID-19 Patients Admitted to Health Facilities in Sierra Leone in 2020–2021: Practice Does Not Follow Policy

**DOI:** 10.3390/ijerph19074005

**Published:** 2022-03-28

**Authors:** Ibrahim Franklyn Kamara, Ajay M. V. Kumar, Anna Maruta, Bobson Derrick Fofanah, Charles Kuria Njuguna, Steven Shongwe, Francis Moses, Sia Morenike Tengbe, Joseph Sam Kanu, Sulaiman Lakoh, Alie H. D. Mansaray, Kalaiselvi Selvaraj, Mohammed Khogali, Rony Zachariah

**Affiliations:** 1 World Health Organization, 21A-B Riverside, Off King Harman Road Freetown, Freetown 00232, Sierra Leone; marutaa@who.int (A.M.); fofanahb@who.int (B.D.F.); njugunach@who.int (C.K.N.); shongwes@who.int (S.S.); 2 International Union Against Tuberculosis and Lung Disease, 68 Boulevard Saint Michel, 75006 Paris, France; akumar@theunion.org; 3 International Union Against Tuberculosis and Lung Disease, South-East Asia Office, C-6 Qutub Institutional Area, New Delhi 110016, India; 4 Yenepoya Medical College, Yenepoya (Deemed to be University), University Road, Deralakatte, Mangalore 575018, India; 5 Ministry of Health and Sanitation, 4th Floor, Youyi Building, Brookfields, Freetown 00232, Sierra Leone; franqoline@gmail.com (F.M.); siamoreniketengbe@outlook.com (S.M.T.); lakoh2009@gmail.com (S.L.); 6 National Disease Surveillance Program, Ministry of Health and Sanitation, Sierra Leone National Public Health Emergency Operations Centre, Cockerill, Wilkinson Road, Freetown 00232, Sierra Leone; samjokanu@yahoo.com; 7 Department of Community Health, Faculty of Clinical Sciences, College of Medicine and Allied Health Sciences, University of Sierra Leone, Freetown 00232, Sierra Leone; 8 Crops Division, Ministry of Agriculture and Forestry, Youyi Building, Brookfields, Freetown 00232, Sierra Leone; aliemans270@gmail.com; 9 All India Institute of Medicine Sciences, Nagpur 441108, India; kalaiselvi@aiimsnagpur.edu.in; 10 Special Program for Research and Training in Tropical Diseases (TDR), World Health Organization, Avenue Appia 20, 1211 Geneva, Switzerland; khogalim@who.int (M.K.); zachariahr@who.int (R.Z.)

**Keywords:** antimicrobial resistance, COVID-19, AWaRe classification, antimicrobial stewardship, antibiotic use, SORT IT, operational research, Sierra Leone

## Abstract

Inappropriate use of antibiotics during the COVID-19 pandemic has the potential to increase the burden of antimicrobial resistance. In this study, we report on the prevalence of antibiotic use and its associated factors among suspected and confirmed COVID-19 patients admitted to 35 health facilities in Sierra Leone from March 2020–March 2021. This was a cross-sectional study using routinely collected patient data. Of 700 confirmed COVID-19 patients, 47% received antibiotics. The majority (73%) of the antibiotics belonged to the ’WATCH’ group of antibiotics, which are highly toxic and prone to resistance. The most frequently prescribed antibiotics were azithromycin, ceftriaxone, amoxicillin, metronidazole, and amoxicillin-clavulanic acid. Antibiotic use was significantly higher in patients aged 25–34 years than in those with severe disease. Of 755 suspected COVID-19 patients, 61% received antibiotics, of which the majority (58%) belonged to the ‘WATCH’ category. The most frequently prescribed antibiotics were ceftriaxone, metronidazole, azithromycin, ciprofloxacin, and amoxycillin. The prevalence of antibiotic use among suspected and confirmed COVID-19 patients admitted to healthcare facilities in Sierra Leone was high and not in line with national and WHO case management guidelines. Training of health care providers, strengthening of antimicrobial stewardship programs, and microbiological laboratory capacity are urgently needed.

## 1. Introduction

Antimicrobial resistance (AMR) is a global public health concern that has the potential to reverse decades of progress in decreasing the morbidity and mortality from infectious disease outbreaks and pandemics [1,2,3]. In 2019 alone, 1.27 million deaths were attributed to bacterial AMR, with the highest age-adjusted death rates in western sub-Saharan Africa. This mortality burden is similar to the global HIV deaths (680,000) and malaria deaths (627,000) combined and ranks behind only the coronavirus disease 2019 (COVID-19) and tuberculosis in terms of global deaths from infection [4]. The number of deaths due to AMR is estimated to increase to about 10 million annually by 2050 if no actions are taken [5].

The use of antimicrobial agents (including appropriate use, inappropriate use, overuse, misuse, and underuse) drives the development and spread of AMR [6,7,8]. Several studies have confirmed that AMR rates are higher in countries that use antimicrobial drugs more often [9,10].

It has been documented that approximately one-third of patients admitted to healthcare facilities receive antimicrobial agents during their hospital stay [11,12]. More alarming is the fact that up to 50% of all courses of antimicrobial therapy are deemed unnecessary [13,14,15]. The misuse of antimicrobial agents (antibiotics) will increase multi-drug resistance (MDR) with higher mortality, longer hospital stays, and increased costs to both the patients and the hospital management [16]. This is a growing threat to the effective treatment of an increasing range of infectious disease outbreaks, such as the ongoing COVID-19 pandemic [17,18,19].

COVID-19 was declared by the World Health Organization (WHO) to be a global pandemic on 11 March 2020, and since then the disease has had devastating health and economic consequences [20,21]. As of date, there are limited treatment options for COVID-19 patients. The treatment options available include supportive care, invasive and non-invasive oxygen support, anticoagulants, and the use of systemic corticosteroids in severe and critical patients, which has been shown to prevent deaths due to COVID-19 [22,23]. While many vaccines are now approved for use, equitable access remains a challenge.

At the start of the COVID-19 outbreak in Wuhan city, Hubei province, China, in December 2019, 90% of hospitalized COVID-19 patients at their healthcare facilities received antibiotics despite little supporting evidence of associated bacterial infections [24]. The realization of the inappropriate management of COVID-19 patients led the WHO to develop clinical guidelines for managing COVID-19 patients. The WHO case management clinical guidelines recommend that people with suspected COVID-19 (while being investigated) should not be treated with antibiotics. Similarly, people with laboratory-confirmed COVID-19 who are asymptomatic or have mild symptoms should not receive antibiotic treatment or prophylaxis. Patients with moderate or severe COVID-19 should not be given antibiotics unless there is clinical suspicion of a bacterial infection, while critically ill COVID-19 patients should receive antibiotics within an hour of admission [23]. It is further recommended that the choice of antibiotic be based on clinical diagnosis, local epidemiology, antibiotic susceptibility data, and national guidelines. It is preferable to use an antibiotic with the least ecological impact, such as from the ‘access’ group of the WHO AWaRe classification of antibiotics [23]. However, despite the presence of WHO case management clinical guidelines, there is growing concern about the misuse of antibiotics in the treatment of COVID-19 patients [19,25,26].

A recent rapid review and meta-analysis that involved 154 studies globally reported that the prevalence of antibiotic use among COVID-19 patients was 74.6%, while only 8.6% had bacterial co-infection—indicating high rates of unnecessary antibiotic use [27]. Antibiotic use was higher in adults (compared to children) and in those with more severe illnesses, such as those hospitalized and requiring mechanical ventilation. The most common antibiotic classes prescribed were fluoroquinolones, macrolides, β-lactam/β-lactamase inhibitors, and cephalosporins. The prevalence of antibiotic use was higher in the Southeast Asia region and the Middle East compared to the Americas and Europe [25].

Despite the need to monitor and document antibiotic use in COVID-19 patients, there is limited evidence from Africa on the use of antibiotics among suspected and confirmed COVID-19 patients, and there are no studies on this issue from Sierra Leone. Studies documenting the use of antibiotics in COVID-19 have the potential to inform national policies and can help to design antibiotic stewardship programs in future outbreaks and the choice of antibiotics in routine patient care. Furthermore, findings from this study will add to the global body of evidence on the use of antibiotics in the management of viral infections. The aim of the study, therefore, was to assess antibiotic use among suspected and confirmed COVID-19 patients in Sierra Leone. The specific objectives were the following: (i) to assess the prevalence of antibiotic use, (ii) to classify prescribed antibiotics according to the WHO antibiotic AWaRe classification, and (iii) to determine demographic and clinical factors associated with antibiotic use among suspected and confirmed COVID-19 patients admitted to health facilities and community care centres in Sierra Leone from 31 March 2020 to 31 March 2021.

## 2. Materials and Methods

### 2.1. Study Design

This was a cross-sectional study involving secondary analysis of routinely collected patient data.

### 2.2. Study Setting

Sierra Leone is a country in West Africa bordered by Guinea, Liberia, and the Atlantic Ocean and is divided into 16 districts. The estimated population is 8 million and most (59%) of the people live in rural areas [28]. In 2018, the life expectancy was 53 years for males and 55 years for females, with communicable diseases accounting for 57% of all-cause mortality [25]. The total expenditure on health as a percentage of gross domestic product for Sierra Leone in 2020 was 16% [25].

### 2.3. Diagnosis and Management of COVID-19 Patients

Individuals suspected of COVID-19 infection (defined as any person presenting with a history of fever ≥ 37.5 °C and at least one sign/symptom of respiratory disease, e.g., cough, shortness of breath, sore throat, loss of smell, fatigue) visiting health facilities are usually admitted to the ‘isolation units’ and investigated for COVID-19 using RT-PCR (reverse transcriptase-polymerase chain reaction) laboratory tests. Asymptomatic patients were admitted during the study period to reduce community transmission. Once confirmed as COVID-19 positive, the patient is referred to the ‘COVID-19 treatment centres’ or ‘community care centres’. Sierra Leone has a total of 21 centres (18 treatment and 3 community care centres) for the management of confirmed COVID-19 patients across the country. The ‘COVID-19 treatment centres’ provide care for moderate, severe, and critical COVID-19 patients whereas ‘community care centres’ are structures (such as schools or university buildings) or healthcare facilities repurposed to provide care to asymptomatic patients and those with mild disease. COVID-19 treatment is offered free of cost to the patients. According to the policy in Sierra Leone, medical doctors and community health officers are responsible for the prescription of antimicrobial agents. The national case management guidelines, including antibiotic use in Sierra Leone, follow WHO guidelines (Table 1).

### 2.4. AWaRe Classification

The AWaRe classification of antibiotics is an excellent tool to prevent AMR and strengthen antimicrobial stewardship at local, national, and global levels. AWaRe classifies antibiotics into the following three groups: ACCESS, WATCH, and RESERVE groups [26]. The ‘ACCESS’ group of antibiotics are those which should always be available as they are used in the treatment of common bacterial infections, and they show lower resistance potential [29]. The ‘WATCH’ group of antibiotics includes most of the highest priority agents among the critically important antimicrobials. They have higher resistance potential and are the key targets for antimicrobial stewardship programmes and monitoring [30]. The ‘RESERVE’ group should be treated as ‘last resort’ antibiotics to be used in the management of multidrug-resistant infections, and they are a key target for national and international antimicrobial stewardship programs [31].

### 2.5. Study Population and Period

We included all the people with suspected and confirmed COVID-19 infection admitted to community care centres, treatment centres, and isolation units between 31st March 2020 and 31 March 2021. For the study of confirmed COVID-19 patients, all the community care centres (*n* = 3) and treatment centres (*n* = 18) in the country were included. For the study of suspected COVID-19 patients, we included isolation units (*n* = 14) in secondary and tertiary healthcare facilities in the country. There was no sampling, and we reviewed all the patient records that were present at the study sites from the day of the commissioning of these designated units to the time of data collection.

### 2.6. Data Variables, Data Sources, and Data Collection

Data variables included age, sex, location of the centre (rural or urban), COVID-19 case type (suspected or confirmed), the severity of disease at the time of admission (asymptomatic, mild, moderate, severe, and critical), duration of admission, antibiotic use (yes or no), and if ‘yes’, the name of the antibiotic used. A paper-based, structured data collection proforma was used to collect data after pre-testing and validation. The primary source of data was the patient chart (booklet) that contained details of the treatments (including antibiotic use) and other medical care given to the patient at the COVID-19 isolation units, community care, and treatment centres. Data collection was performed by the principal investigator and four data collectors, who were trained before data collection for this purpose from 31 May to 31 August 2021.

### 2.7. Data Entry and Analysis

To ensure the highest standards in data quality, we performed double entry and validation using EpiData (version 3.1, EpiData Association, Odense, Denmark). Data analysis was performed using EpiData analysis (v2.2.2.187) and Stata (v16, StataCorp, College Station, TX, USA) software.

Data regarding demographic and clinical characteristics were summarized using frequencies and proportions. Duration of hospital stay among confirmed COVID-19 patients was presented as the median number of days with an interquartile range. Analysis was conducted separately for suspected COVID-19 and confirmed COVID-19 patients. Prevalence of antibiotic use was reported as a percentage with 95% confidence intervals (CI). The pattern of antibiotic use was described according to the WHO AWaRe classification.

We assessed associations of demographic and clinical characteristics with antibiotic use using log-binomial regression. We calculated adjusted prevalence ratios (and 95% CI) by including all the variables in the multivariable model in line with our exploratory approach. In all analyses, a *p*-value ≤ 0.05 was considered statistically significant.

## 3. Results

### 3.1. Patients with Confirmed COVID-19

The demographic and clinical characteristics of COVID-19 patients are described in Table 2. There were a total of 700 confirmed COVID-19 patients, of whom 406 (58%) were males and nearly two-thirds (64%) were from rural areas. The median (IQR) age of patients was 35 (25–52) years. About 85% of all admitted patients were classified as asymptomatic or mild, and the median duration of admission was 13 (10–18) days. There were no critical cases of COVID-19.

**Figure 1:**
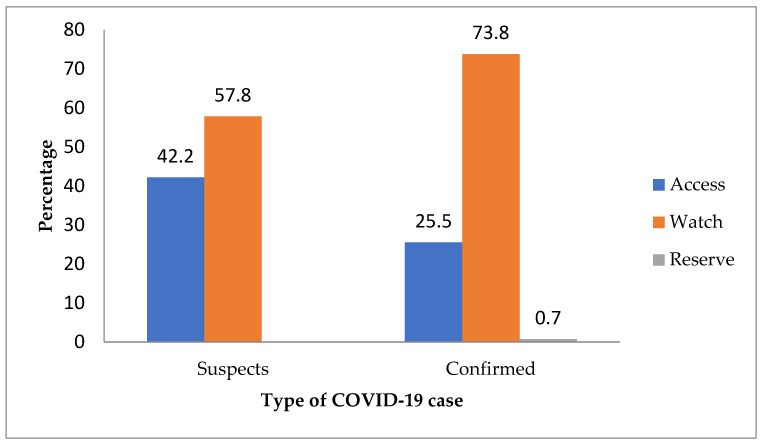
Prescription of antibiotics according to the WHO AWaRe classification of antibiotic use in suspected and confirmed COVID-19 patients admitted to isolation units and treatment centres in Sierra Leone (March 2020–March 2021).

**Figure 2:**
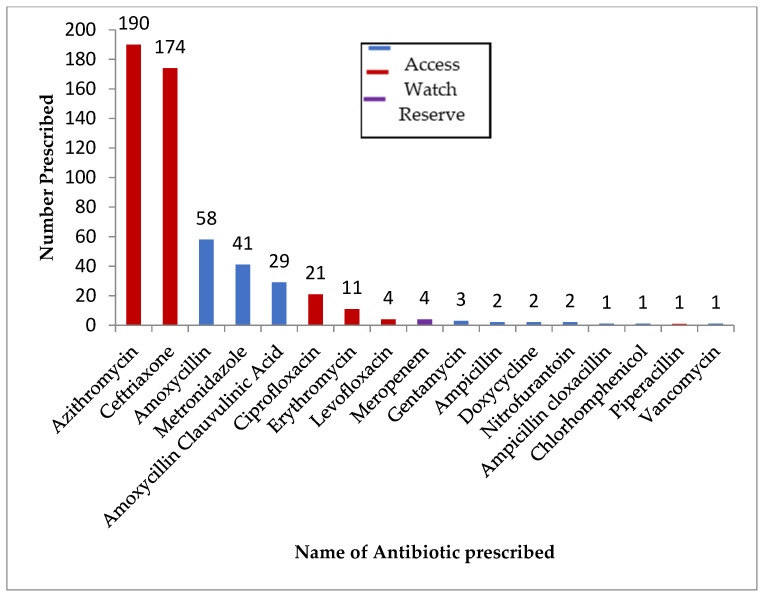
Different antibiotics prescribed to COVID-19 confirmed patients admitted to community care and treatment centres in Sierra Leone, March 2020–March 2021 (*N* = 545).

The prevalence of antibiotic use among confirmed COVID-19 patients was 47% (95% CI: 43–51%). The prevalence of antibiotic use ranged from 32% to 100% across the districts among COVID-19 confirmed patients. The majority (73%) of the antibiotics prescribed fell under ‘WATCH’ category of the WHO antibiotic AWaRe classification, and a total of four patients were prescribed ‘RESERVE’ category antibiotics (meropenem) as shown in Figure 1. The median number of antibiotics prescribed to a patient was 2 (IQR: 1–2), and the maximum number was six. The most frequently prescribed antibiotics were azithromycin, ceftriaxone, amoxycillin, metronidazole, and amoxycillin-clavulanic acid (Figure 2).

The prevalence of antibiotic use was significantly higher in people with mild (PR: 2.0, 95% CI: 1.8–2.7), moderate (PR: 2.1, 95% CI: 1.5–2.8) and severe disease (PR: 2.2, 95% CI: 1.9–2.9) as compared to asymptomatic patients. There was no significant difference in antibiotic use between mild, moderate, and severe patients. In an unadjusted analysis, we found that patients in the 25–34 year age group had a lower prevalence (PR: 0.7, 95% CI: 0.5–1.0) and the older age group had a higher prevalence of antibiotic use (PR: 1.9, 95% CI: 1.4–2.6) compared to children. Patients in urban areas had a higher prevalence (PR: 1.9, 95% CI: 1.6–2.2) of antibiotic use compared to those in rural areas. Duration of admission and sex were not associated with antibiotic use. In adjusted analysis, only age (25–34 years) and disease severity (mild, moderate, and severe) emerged as independent predictors of antibiotic use (Table 3).

There were a total of 755 suspected COVID-19 patients, with the majority (74%) from the urban area. Of these patients, 369 (49%) were males, and the median (IQR) age was 33 (25–45) years (Table 4).

### 3.2. Patients with Suspected COVID-19

**Figure 3:**
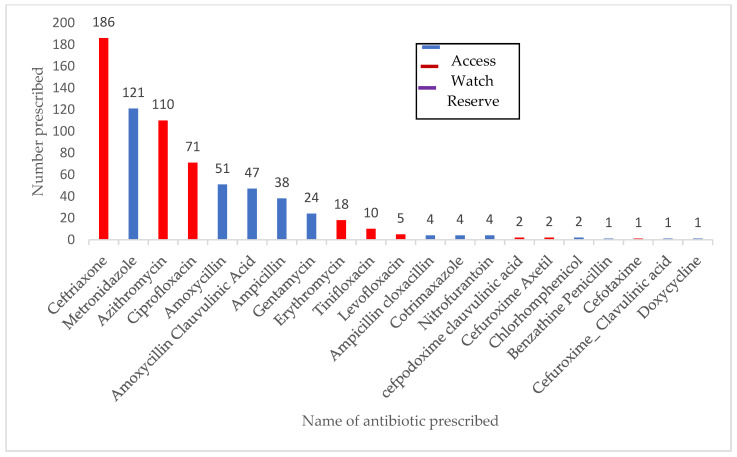
Different antibiotics prescribed to COVID-19 suspected patients admitted to isolation units in Sierra Leone, March 2020–March 2021 (*N* = 703).

The prevalence of antibiotic use among suspected COVID-19 patients was 61% (95% CI: 58–65%) and the majority (58%) of the antibiotics prescribed fell under the ‘WATCH’ category, as shown in Figure 1 and Figure 3. The prevalence of antibiotic use ranged from 55% to 94% across the districts among COVID-19 suspects. The median number of antibiotics prescribed to a patient was two (one–two); the minimum number prescribed was one, and the maximum number was six. The most frequently prescribed antibiotics were ceftriaxone, metronidazole, azithromycin, ciprofloxacin, and amoxycillin, respectively (Figure 3).

Adjusted analysis showed that the prevalence of antibiotic use was significantly lower in urban areas compared to rural areas, and older age groups had a higher prevalence of antibiotic use compared to children (Table 5).

## 4. Discussion

This is the first study from Sierra Leone reporting on the prevalence of antibiotic use and its associated factors among suspected and confirmed COVID-19 patients admitted to healthcare facilities. This adds to the global evidence on the use of antibiotics in the management of COVID-19. It further contributes to the evidence of inappropriate use of antimicrobial agents globally, which has the potential to increase antimicrobial resistance. There were three key findings, and we will discuss them below.

First, nearly half of all confirmed COVID-19 patients received antibiotics in Sierra Leone. Most (~85%) of the confirmed COVID-19 patients were either ‘asymptomatic’ or had a mild illness and should not have received antibiotics. About six in ten suspected COVID-19 patients received antibiotics when none should have received them as per the WHO and national case management guidelines. In our view, such high levels of antibiotic use were unnecessary and not in line with both the national and WHO clinical case management guidelines. In addition to the misuse of antibiotics among COVID-19 patients, our study provided an insight into the prescribing practises of clinicians which is relevant to the establishment of antimicrobial stewardship programmes in health care facilities. However, despite these worrying results, they are similar to reports from other countries in the African Region (47% in Kenya, 71% in South Africa, and 76% in Uganda) and elsewhere (78% in Spain, 83% in the USA, 100% in Bangladesh, and 67–90% in several studies from China) showing high use of antibiotics in COVID-19 patients [32,33,34,35,36,37,38,39]. In contrast, a study from Singapore reported a very low level (~5%) of antibiotic use [40]. Most of the studies have focused on antibiotic use among confirmed COVID-19 patients and not in suspected patients. Our study is the first from Africa reporting on suspected COVID-19 patients, and we add to the limited evidence for this group of patients. The only other study that looked at suspected COVID-19 patients was from Singapore, which reported a prevalence of 39% antibiotic use [40]. This inappropriate use of antibiotics during the ongoing COVID-19 pandemic will increase multidrug resistance and be associated with longer hospital stays and increased costs to both patients and hospital management.

Second, the majority of patients in our study received at least two antibiotics, with the most predominant antibiotics being azithromycin, ceftriaxone, amoxycillin, metronidazole, and amoxycillin-clavulanic acid. Most of these antibiotics fall under the ‘WATCH’ group of drugs according to the WHO AWaRe categorization. This is in contrast to the national and WHO guidelines which recommend using the ‘ACCESS’ group of antibiotics where warranted and restricting the use of ‘WATCH’ and ‘RESERVE’ groups of antibiotics. There has been a lot of inconsistency in the normative guidance available, adding to the confusion regarding the choice of antibiotics in COVID-19 patients. A rapid review of national treatment guidelines for COVID-19 in 10 African countries showed that various antibiotics, such as azithromycin, doxycycline, clarithromycin, ceftriaxone, erythromycin, amoxicillin, amoxicillin-clavulanic acid, ampicillin, gentamicin, benzylpenicillin, piperacillin/tazobactam, ciprofloxacin, ceftazidime, cefepime, vancomycin, meropenem, and cefuroxime, among others, were recommended for use in the management of COVID-19 [41]. Most of these antibiotics were from the ‘WATCH’ and ‘RESERVE’ categories, in contrast to the WHO guidelines.

Third, severity of disease and age were independent predictors of antibiotic use among confirmed COVID-19 patients. In suspected COVID-19 patients, adults were more likely to receive antibiotics compared to children, and patients admitted in rural areas were more likely to receive antibiotics compared to those in urban areas. These findings are similar to reports from other settings. The study from Bangladesh reported that patients presenting with severe disease (especially those with co-morbidities such as diabetes mellitus) received more antibiotics on average [35]. The study from Spain reported that inappropriate antibiotic use was more likely in patients in the younger age groups and in those without co-morbidities [36]. Similarly, a study from USA reported an association of antibiotic use with increased duration of hospital stay and with patients admitted to ICU and needing mechanical ventilation [37]. In contrast, we did not find any association between the duration of a hospital stay and antibiotic use. The study from Singapore reported that antibiotic use was more appropriate when it was prescribed by infectious disease physicians [40].

Our study had several strengths. First, it was a countrywide study with a large sample size, and, therefore, the findings are representative of all COVID-19 patients and suspects who were admitted to health care facilities in Sierra Leone. Second, data collection was performed by clinicians who were well versed in reading patient clinical files. Third, we used a structured data collection proforma that was pretested and validated before use. This facilitated the implementation of uniform procedures in data collection. Finally, we adhered to ‘STROBE’ (Strengthening the Reporting of Observational Studies in Epidemiology) guidelines for reporting the study findings.

There were some limitations. The first relates to the study population. As of 31 March 2021, Sierra Leone had reported a total of 3964 confirmed COVID-19 patients. We did not include COVID-19 patients who received home care or any other treatment options. Thus, we will not be able to extrapolate our findings to these patients. This requires future research. The second limitation relates to the lack of information on the prevalence of bacterial co-infection in our study, which made it difficult for us to make a judgement on the appropriateness of antibiotic use in individual patients. Nevertheless, studies globally have reported a pooled low prevalence of 8.6% of bacterial co-infections in COVID-19 patients, and we have no reason to believe that Sierra Leone will be any different [42]. The third limitation was that we did not look at variation in antibiotic use among suspected and confirmed COVID-19 patients during the different phases of the ongoing COVID-19 pandemic in the country. Despite these limitations, there are some important policy and practice implications arising out of the study.

Inappropriate use of antibiotics has many implications, which include increased costs of health care, increased incidence of adverse drug events, increased mortality, and an increase in antimicrobial resistance in the long run, making antibiotic treatments ineffective when needed. A study from Spain reported that the incidence of adverse drug events was four times higher in COVID-19 patients who received antibiotics inappropriately compared to those with appropriate antibiotic use [36]. Possible reasons for inappropriate use of antibiotics in COVID-19 patients include (i) lack of an effective antibiotic stewardship programme in the health care facilities, (ii) lack of knowledge about the WHO and national treatment guidelines and AWaRe classification, (iii) lack of access to rapid diagnostics leading to empirical antibiotic use, and (iv) lack of supervision, monitoring, and review. It is also possible that clinicians in the early stages of the pandemic used antibiotics as a ‘safety net,’ in the absence of specific and effective antivirals to treat COVID-19.

We make the following recommendations to address the overuse of antibiotics in COVID-19 patients in Sierra Leone. First, all healthcare workers should be trained in national case management guidelines, including when to use antibiotics, what antibiotics to be used, and the AWaRe classification. Although the national guidelines have been developed, they have not been widely disseminated. This is of the utmost priority. Second, antimicrobial stewardship programmes should be established and strengthened at all the hospitals in Sierra Leone. This includes having dedicated focal points and teams reviewing antibiotic use in the respective health care facilities by conducting periodic point prevalence surveys (once every three or six months) to monitor the situation and take appropriate action. Third, access to quality-assured rapid diagnostics, including culture and sensitivity testing and molecular technologies, should be strengthened. This will obviate the need for empirical treatment and promote a more rational use of antibiotics.

## 5. Conclusions

In this first study from Sierra Leone, we found that there was a high prevalence of antibiotic use among suspected (60%) and confirmed COVID-19 (48%) patients admitted to the health care facilities and community care centres. Since 85% of the confirmed COVID-19 patients were asymptomatic or had mild illnesses, they did not need antibiotics. Similarly, about 60% of suspected COVID-19 patients received antibiotics, when none should have received them as per the guidelines. Such high levels of antibiotic use were unjustified and were not in line with the national and global guidelines. The most frequently prescribed antibiotics were from the ‘Watch’ group of the AWaRe categorisation, which are toxic and prone to resistance. The most frequently prescribed antibiotics were azithromycin, amoxycillin, ceftriaxone, and metronidazole. These issues need to be addressed urgently because inappropriate use of antibiotics leads to increased costs of health care, an increased incidence of adverse drug events, and increased antimicrobial resistance in the long run. We recommend that (i) healthcare workers be trained on national case management guidelines, including the use of antibiotics; (ii) antimicrobial stewardship programmes be strengthened at hospitals; (iii) laboratory capacities be improved to conduct culture and antibiotic sensitivity testing.

## Figures and Tables

**Table 1:** COVID-19 disease classification, signs and symptoms, and antibiotic use according to the 2020 WHO clinical management guidelines.

Disease Severity	Signs and Symptoms	Antibiotic Use
Asymptomatic	No symptoms	No
Mild	Fever, cough, fatigue, anorexia, shortness of breath, myalgia	No
Moderate	Fever, cough, dyspnoea, fast breathing, SpO2 ≥ 90% on room air	Yes, only if suspicion of bacterial infection
Severe	Fever, cough, dyspnoea, fast breathing, respiratory rate > 30 breaths/min, severe respiratory distress, SpO2 < 90% on room air	Yes, only if suspicion of bacterial infection
Critical	Lobar or lung collapse, respiratory failure, PaO2/FiO2a ≤ 300 mmHg- PaO2/FiO2 ≤ 100 mmHg, acute life-threatening organ dysfunction, fast heart rate, weak pulse, cold extremities or low blood pressure, skin mottling, coagulopathy, thrombocytopenia, acidosis, high lactate, or hyperbilirubinemia.	Yes, within one hour of admission

SpO2—Oxygen saturation; PaO2—Partial pressure of oxygen; FiO2—Fraction of inspired oxygen.

**Table 2:** Demographic and clinical characteristics of people with confirmed COVID-19 infection admitted in community care and treatment centres in Sierra Leone, March 2020–March 2021 (*N* = 700).

Variable	*N*	(%)
Region		
Urban	254	(36.3)
Rural	446	(63.7)
Sex		
Male	406	(58.0)
Female	288	(41.1)
Missing	6	(0.9)
Age (years)		
0–14	62	(8.9)
15–24	103	(14.7)
25–34	165	(23.6)
35–44	118	(16.9)
45–54	95	(13.6)
55–64	74	(10.6)
≥65	80	(11.4)
Missing	3	(0.4)
Disease classification		
Asymptomatic	441	(63.0)
Mild	160	(22.9)
Moderate	24	(3.4)
Severe	65	(9.3)
Missing	10	(1.4)
Duration of admission		
<7 days	116	(16.6)
7–14 days	283	(40.5)
>14 days	299	(42.8)

**Table 3:** Prevalence of antibiotic use and its associated factors among people with confirmed COVID-19 infection admitted in community care centres and treatment centres of Sierra Leone, March 2020–March 2021 (*N* = 700).

Variable	Total	Antibiotic Use *N*	(%)	PR	(95% CI)	aPR	95% CI
Total							
Region							
Urban	254	172	(67.7)	1.91	(1.6–2.2)	1.19	(1.0–1.5)
Rural	446	158	(35.4)	Ref	Ref	Ref	Ref
Sex							
Male	406	203	(50.0)	Ref	Ref	Ref	Ref
Female	288	126	(43.8)	0.88	(0.7–1.0)	1.02	(0.9–1.1)
Age (years)							
0–14	62	26	(41.9)	Ref	Ref	Ref	Ref
15–24	103	45	(43.7)	1.04	(0.7–1.5)	0.97	(0.7–1.4)
25–34	165	49	(29.7)	0.71	(0.5–1.0)	0.64 *	(0.4–0.9)
35–44	118	45	(38.1)	0.91	(0.6–1.3)	0.75	(0.5–1.1)
45–54	95	52	(54.7)	1.31	(0.9–1.8)	0.86	(0.6–1.2)
55–64	74	49	(66.2)	1.58	(1.1–2.2)	0.95	(0.7–1.4)
≥65	80	64	(80.0)	1.91	(1.4–2.6)	1.03	(0.7–1.5)
Disease classification							
Asymptomatic	441	136	(30.8)	Ref	Ref	Ref	Ref
Mild	160	121	(75.6)	2.45	(2.1–2.9)	2.00 *	(1.8–2.7)
Moderate	24	16	(66.7)	2.16	(1.6–3.0)	2.05 *	(1.5–2.8)
Severe	65	54	(83.1)	2.69	(2.3–3.2)	2.16 *	(1.9–2.9)
Duration of admission							
<7 days	116	66	(56.9)	Ref	Ref	Ref	Ref
7–14 days	283	121	(42.8)	0.75	(0.6–0.9)	0.91	(0.7–1.1)
>14 days	299	141	(47.2)	0.83	(0.1–0.7)	0.97	(0.8–1.2)

PR–Prevalence ratio; CI–confidence intervals; aPR–adjusted prevalence ratio; * statistically significant (*p* value < 0.05).

**Table 4:** Demographic characteristics of people with suspected COVID-19 infection admitted to isolation units in Sierra Leone, March 2020–March 2021 (*N* = 755).

Variable	*N*	(%)
Location		
Urban	584	(77.4)
Rural	171	(22.6)
Sex		
Male	369	(48.9)
Female	385	(51)
Missing	1	(0.1)
Age (years)		
0–14	67	(8.9)
15–24	119	(15.8)
25–34	205	(27.2)
35–44	161	(21.3)
45–54	94	(12.4)
55–64	49	(6.5)
≥65	59	(7.8)
Missing	1	(0.1)

**Table 5:** Prevalence of antibiotic use and its associated factors among people with suspected COVID-19 infection admitted in isolation units in Freetown, Sierra Leone, March 2020–March 2021 (*N* = 755).

Variable	Total	Antibiotic Use *N*	(%)	PR	95% CI	aPR	95% CI
Location							
Urban	584	319	(54.6)	0.65	(0.6–0.7)	0.67 *	(0.6–0.7)
Rural	171	144	(84.2)	Ref	Ref	Ref	Ref
Sex							
Male	369	233	(63.1)	Ref	Ref	Ref	Ref
Female	385	229	(59.5)	0.94	(0.8–1.1)	0.97	(0.9–1.1)
Age (years)							
0–14	67	26	(38.8)	Ref	Ref	Ref	Ref
15–24	119	75	(63)	1.62	(1.2–2.3)	1.54 *	(1.1–2.1)
25–34	205	134	(65.4)	1.68	(1.2–2.3)	1.56 *	(1.1–2.1)
35–44	161	93	(57.8)	1.49	(1.1–2.1)	1.41 *	(1.0–1.9)
45–54	94	61	(64.5)	1.67	(1.2–2.3)	1.52 *	(1.1–2.1)
55–64	49	33	(67.4)	1.73	(1.2–2.5)	1.63 *	(1.2–2.3)
≥65	59	41	(69.5)	1.79	(1.3–2.5)	1.55 *	(1.1–2.2)

PR—Prevalence ratio; CI—confidence intervals; aPR—adjusted prevalence ratio; * statistically significant (*p* value < 0.05).

## Data Availability

The dataset used in this paper has been deposited at https://doi.org/10.6084/m9.figshare.19158734 (accessed on 11 February 2022) and is available under a CC BY 4.0 license.
